# Drought stress amelioration in tomato (*Solanum lycopersicum* L.) seedlings by biostimulant as regenerative agent

**DOI:** 10.3389/fpls.2023.1211210

**Published:** 2023-08-15

**Authors:** Metin Turan, Melek Ekinci, Sanem Argin, Mihail Brinza, Ertan Yildirim

**Affiliations:** ^1^ Department of Agricultural Trade and Management, Faculty of Economy and Administrative Sciences, Yeditepe University, Istanbul, Türkiye; ^2^ Department of Horticulture, Faculty of Agriculture, Atatürk University, Erzurum, Türkiye; ^3^ Humintech GmbH, Grevenbroich, Germany; ^4^ Atatürk University Plant Production Application and Research Center, Erzurum, Türkiye

**Keywords:** biostimulant, drought stress, growth, physiology, *Solanum lycopersicum*

## Abstract

Drought adversely affects many physiological and biochemical events of crops. This research was conducted to investigate the possible effects of biostimulants containing plant growth-promoting rhizobacteria (PGPR) on plant growth parameters, chlorophyll content, membrane permeability (MP), leaf relative water content (LRWC), hydrogen peroxide (H_2_O_2_), proline, malondialdehyde (MDA), hormone content, and antioxidant enzymes (catalase (CAT), peroxidase (POD), and superoxide dismutase (SOD)) activity of tomato (*Solanum lycopersicum* L.) seedlings under different irrigation levels. This study was carried out under controlled greenhouse conditions with two irrigation levels (D0: 100% of field capacity and D1: 50% of field capacity) and three biostimulant doses (B0: 0, B1: 4 L ha^-1^, and B2: 6 L ha^-1^). The results of the study show that drought stress negatively influenced the growth and physiological characteristics of tomato seedlings while biostimulant applications ameliorated these parameters. Water deficit conditions (50% of field capacity) caused decrease in indole acetic acid (IAA), gibberellic acid (GA), salicylic acid (SA), cytokine, zeatin, and jasmonic acid content of tomato seedlings by ratios of 83%, 93%, 82%, 89%, 50%, and 57%, respectively, and shoot fresh weight, root fresh weight, shoot dry weight, root dry weight, plant height, stem diameter, and leaf area decreased by 43%, 19%, 39%, 29%, 20%, 18%, and 50%, respectively, compared to the control (B0D0). In addition, 21%, 16%, 21%, and 17% reductions occurred in LRWC, chlorophyll a, chlorophyll b, and total chlorophyll contents with drought compared to the control, respectively. Biostimulant applications restored the plant growth, and the most effective dose was 4 L ha^-1^ under drought condition. Amendment of biostimulant into the soil also enhanced organic matter and the total N, P, Ca, and Cu content of the experiment soil. In conclusion, 4 L ha^-1^ biostimulant amendment might be a promising approach to mitigate the adverse effects of drought stress on tomato.

## 1 Introduction

Among the natural disasters with meteorological character, the one with the most comprehensive effect is drought. Drought causes significant social, environmental, and economic damage. Arid and semi-arid regions in the world are estimated to comprise 16% of the total land, which corresponds to around 21-22 million km^2^. In such regions, factors such as lack of precipitation, irregularity of precipitation regimes, and scarcity of resources are among the main problems of agriculture. In order to solve the problem of drought, especially due to water scarcity and lack of precipitation, and to bring agriculture into these regions, either irrigation activities are developed or the application of dry farming methods is attempted (Oweis et al., 1999; Ekinci et al., 2015). Climate change further reduces the water availability, efficiency, and causes drought stress in major agro-systems of the world, especially in rain-fed ecosystems (FAO, 2020). In particular, water scarcity is a very important factor for the yield of vegetable crops. Drought adversely affects the root growth, root length, water nutrient uptake and transport, photosynthetic activity, seed germination, seedling emergence, carbon assimilation, dry matter accumulation, flowering, pollen formation, fertilization, and, ultimately, the yield of vegetable crops (Ors et al., 2016; Nephali et al., 2020; Gedeon et al., 2022).

Foliar, seed, or soil applications of biostimulants increase the resistance of plants against stress, and as a result, plant growth, product quality, and yield are positively affected (Turan et al., 2021; Yildirim et al., 2021a; Niu et al., 2022; Tuver et al., 2022). Biostimulants are plant growth-promoting substances that are both nutrients and soil improvers, such as humic substances, amino acids, hydrolyzed proteins, algae, chitosan-like polymers, inorganic compounds, and beneficial microorganisms (Bulgari et al., 2015; De Vasconcelos and Chaves, 2019; Drobek et al., 2019). Today, the utilization of biostimulants consists of rhizospheric microorganisms, amino acids, humic substances, and enzymes, and it has become an important aspect of sustainable agriculture (Nadeem et al., 2013; Marella, 2014; Adedayo et al., 2022). Mechanisms related to the increase in plant growth caused by biostimulants are divided into two groups: (1) Direct mechanisms: stimulation of growth and plant protection; reduction of plant ethylene synthesis by 1-aminocyclopropane-1-carboxylate deaminase produced in bacteria; improvement of the rhizosphere by the dissolved phosphate and chelated iron by the bacterial activity; and increased plant nutrition by the siderophores produced by the bacteria; (2) Indirect mechanisms: reduction of reactive oxygen species (ROS) through antioxidant production, reduction of osmotic stress, decrease in sodium intake and increased resistance to diseases as a result of chitinase enzyme activity, antibiotic production, and induced systemic resistance (Nadeem et al., 2013). Biostimulants enrich the soil rhizosphere area with provided plant nutrients through N fixation and P and K mineralization, and they promote plant growth with plant growth regulators (Yildirim et al., 2021a).

Tomato (*Solanum lycopersicum*) is an important vegetable crop from the Solanaceae family, native to South and Central America. Turkey is the largest tomato producer after China. However, tomato production does not meet demand as it has been affected by several biotic and abiotic stresses in the last decade (Ali et al., 2021). Climate change, especially water stress, causes a decrease in leaf surface, a reduction in the absorption of minerals, flower shedding, smaller fruits, puffiness, fruit splitting, and calcium deficiency-related disorders such as blossom-end rot (BER) and poor seed viability (Jangid and Dwivedi, 2016). Studies have shown that biostimulants are effective in mitigating the negative impacts of the stress conditions with different mechanisms (Turan et al., 2021).

Many farmers have faced drought stress globally in the last decades. Drought is classified into four distinct categories: meteorological drought that occurs in areas of dry weather; hydrological drought that happens in situations of low and scarce water supply, especially in surface and ground water levels and that is encountered after several months of meteorological drought; agricultural drought that is often associated with decreased soil water levels and consequent crop failures, severely affecting food production all over the world; and the socio-economic drought that relates to failure of supply and demand of various commodities due to drought (Heim, 2002). Among all the sectors, agriculture is most sensitive to water scarcity, and it faces significant decline in yield potential (40 to 60%) in rain-fed areas (FAO, 2020). Therefore, water management is an important task to achieve global food security and zero hunger.

Different crops face different kinds of biotic and abiotic stress. Biotic stress is any stress caused by living organisms such as insects, viruses, bacteria, fungi, and arachnids. Abiotic stress includes conditions such as drought, temperature fluctuations, high soil salinity, metal toxicity, and oxidative stresses. These stresses can cause permanent damage to a plant, such as stunted growth, hampered metabolism, reduced yield, and change in genetic behavior, leading to mutations in the progeny. Drought stress often leads to the accumulation of reactive oxygen species, enzyme inactivation, disrupted membrane structure, damaged ultrastructural cellular components, decreased pistil and pollen development, proline toxicity, hormonal imbalance, reduced photosynthesis, root branching, and root growth, cellular dehydration, decrease in water potential resulting in reduced cell growth, shoot growth, hampered cell expansion and cell wall synthesis, and salt deposition around stomatal openings causing their malfunction.

The aim of this study is to reduce the physiological problems caused by drought stress in the plant and to eliminate yield loss by using new generation biostimulant formulations to increase adaptation and help plants to recover from the stress damage of tomato (*Solanum lycopersicum* L.) seedlings under water deficit conditions.

## 2 Materials and methods

The study was carried out as a pot study under greenhouse conditions, and tomato (*Solanum lycopersicum* L. cv. H2274) was used as the plant material. Throughout the study, the relative humidity was 60-70%, and the temperature was 25 ± 2°C during the day and 18 ± 2°C at night. Tomato seeds were first planted in the peat: perlite (2:1, v:v) was mixed in multi-celled trays, then when the seedlings had 2-3 true leaves, they were transferred to 3 L pots (18 cm diameter and 15 cm high) as one seedling in each pot. The first irrigation for seedlings was performed at field capacity, and the next irrigations were calculated and performed according to the application level (100% and 50%). No fertilizer was applied to the developing seedlings. The pots were filled with the medium prepared with a mixture of soil: peat: sand (2: 1: 1, v: v: v).

A commercial biostimulant product (Microsense^®^ Root) was kindly supplied by Humintech GmbH (Grevenbroich/Germany), and the contents were as follows: 1% Zn, %5 amino acid, % 5 humic substance, and microbial organisms (*Azotobacter chroococcum*, *Azosprillium brasilense*) 1x10^9^ cfu ml^-1^.

Two days after the seedlings were transplanted into pots, biostimulant was applied in 4 L ha^-1^ (B1) and 6 L ha^-1^ (B2) doses as drench in pots. Tap water (B0) was used as the control. The applications were repeated three times with one-week intervals. Subsequent irrigations were continued with tap water (pH: 7.40; EC: 0.20 dS m^-1^).

The amount of water to be given before each irrigation was determined with a portable moisture meter (HH2, Delta-T Devices). The soil water content in each pot was measured before irrigation, and the water required (full irrigation; D0) was determined for the available pot moisture to reach the field capacity applied to the control application. In the water restriction application, the water amount was adjusted according to 50% (D1) of the D0 (100%) application.

The experiment was completed 45 d after transplanting. Shoots and roots were separated for further analysis. At the end of the study, shoot fresh weight, root fresh weight, shoot dry weight, root dry weight, plant height, and stem diameter were measured. For dry weight measurements, the plant material was kept at 70°C for 48 h. To determine the content of proline, hormone, MDA, H_2_O_2_, and antioxidant enzyme activity, roughly 20 g of fresh leaves were frozen in liquid nitrogen and then stored at −80°C. Analyses were performed in quadruplicate.

Electrolyte leakage (EL): 10 leaf discs (10mm in diameter) from the young fully expanded leaves from two plants per replicate were placed in 50 mL glass vials and rinsed with distilled water to remove the electrolytes released during the leaf disc excision. Vials were then filled with 30 ml of distilled water and allowed to stand in the dark for 24 h at room temperature. The EC (EC1) of the bathing solution was determined at the end of the incubation period. Vials were heated in a temperature-controlled water bath at 95°C for 20 min and then cooled to room temperature, and the EC (EC2) was measured. Electrolyte leakage was calculated as a percentage of EC1/EC2 (Shi et al., 2006; Yildirim et al., 2021b).

Leaf relative water content (LRWC): the leaf discs (1 cm in diameter) were cut from randomly selected plants (5 discs for each replicate) and immediately weighed for fresh weight determination (FW). Then, the leaf discs were placed in distilled water for 5 hours to determine the turgor weights. Finally, the discs were oven dried at 72 °C for 48 hours and weighed for dry weight (DW) determination (Yildirim et al., 2021b). Tissue water content was calculated according to the following equation (Arora et al., 1998):


LRWC (%) = [(FW − DW)/(TW − DW)] × 100

Chlorophyll reading values: The amount of chlorophyll was determined by the method defined by Lichtenthaler and Buschmann (2001). The equations below were used to calculate the amounts of chlorophyll a, chlorophyll b, and total chlorophyll in fresh weight (mg g^-1^) (Arnon, 1949; Wellburn, 1994). Where V is the extraction volume and W is the sample weight:


$$\text{Chlorophyll a }\left(\text{mg }{\text{g}}^{-1}\right)\;=\;\left(12.7\;*\;663\text{ nm}\right)\;-\;\left(2.69\;*\;645\text{ nm}\right)\;*\text{ V}/\text{W}*10000$$

$$\text{Chlorophyll b }\left(\text{mg }{\text{g}}^{-1}\right)\;=\;\left(22.91\;*\;645\text{ nm}\right)\;-\;\left(4.68\;*\;663\text{ nm}\right)\;*\text{ V}/\text{W}*10000$$

$$\text{Total chlorophyll }\left(\text{mg }{\text{g}}^{-1}\right)\;=\text{ chlorophyll a }+\text{ chlorophyll b}$$

Leaf area: The total leaf area of a plant was determined by the leaf area meter (CI-202 Portable Laser Leaf Area Meter by CID Bio-Science, USA).

Lipid peroxidation (malondialdehyde-MDA): Lipid peroxidation was defined by the content of MDA. A 0.2 g sample of frozen leaves was grounded to a fine powder with liquid nitrogen and extracted with 3 ml of cold ethanol. The crude extract preparation was centrifuged at 12,000 g for 20 min. A mixture of trichloroacetic acid (TCA), thiobarbituric acid (TBA), butylated hydroxytoluene, and an aliquot of supernatant was heated, and the reaction was stopped quickly by placing the mixture in an ice bath. The cooled mixture was centrifuged, and the absorbance of the supernatant was measured at 400, 500, and 600 nm. Thiobarbituric acid-reactive substances were measured as MDA, a degraded product of the lipid. The concentration of MDA was determined from the absorbance by using an extinction coefficient of 155 mmol l^−1^ cm^−1^ (Shams et al., 2019).

Hydrogen peroxide (H_2_O_2_): H_2_O_2_ was determined according to Velikova et al. (2000). Leaf tissues (200 mg) were homogenized in 2 ml of 0.1% (w/v) TCA solution on ice. The homogenate was centrifuged at 12,000 g for 15 min, and 0.4 ml of the supernatant was added to 0.4 ml of 10 mmol l^−1^ potassium phosphate buffer, pH 7.0, and 0.8 ml of 1 mol l^−1^ KI. The absorbance of the supernatant was measured at 390 nm. The content of H_2_O_2_ was calculated by comparing with a standard calibration curve previously made using different concentrations of H_2_O_2_.

Proline: frozen leaf sample was powdered with liquid nitrogen and extracted with a mortar with sulfosalicylic acid in an ice bath. The homogenates were filtered with a filter paper. Supernatant was reacted with acid ninhydrin and glacial acetic acid in a test tube for 1 h at 100°C, and the reaction terminated in an ice bath. Proline concentration was determined spectrophotometrically at 520 nm (Bates et al., 1973).

CAT, POD, and SOD enzyme activities: Fresh leaf samples were homogenized in the extraction solution according to the method specified by Angelini et al. (1990) and Angelini and Federico (1989), and the obtained supernatant was used to determine enzyme activities. CAT activity was determined by the decrease in absorbance of H_2_O_2_ at 240 nm (Liu et al., 2014). POD activity of the samples was determined at 436 nm and SOD activity at 560 nm with method of Liu et al. (2014) by spectrophotometry.

Hormone analysis: Extraction and purification processes were performed as described by Battal and Tileklioglu (2001) and Kuraishi et al. (1991). Methanol (80%) at –40°C was added to the fresh samples, homogenized at 10 min (Ultra-Turrax, T-25, IKA GmbH &Co), and then incubated for 24 h in dark conditions. Following this, the samples were dried in 35°C and dissolved with 0.1 M KH_2_PO_4_ (pH 8.0). The hormones were determined by HPLC using a Zorbax Eclipse-AAA C-18 column (Agilent 1200 HPLC). Abscisic acid (ABA), cytokinin, gibberellic acid (GA), indole acetic acid (IAA), jasmonic acid, salicylic acid (SA), and zeatin were defined at 265 nm with a UV detector (Ekinci et al., 2014).

Mineral analysis, pH, organic matter (OM), and electrical conductivity (EC) of the soil were determined according to the methods of McLean (1983); Nelson and Sommers (1983), and Rhoades (1983).

Experiments were conducted with a randomized plots design: a total of 108 plants were used with three replications and 6 plants per repeat. The obtained data were subjected to variance analysis using the SPSS statistical program, and the difference between the means was determined by Duncan multiple range tests.

## 3 Results

**Figure 1:**
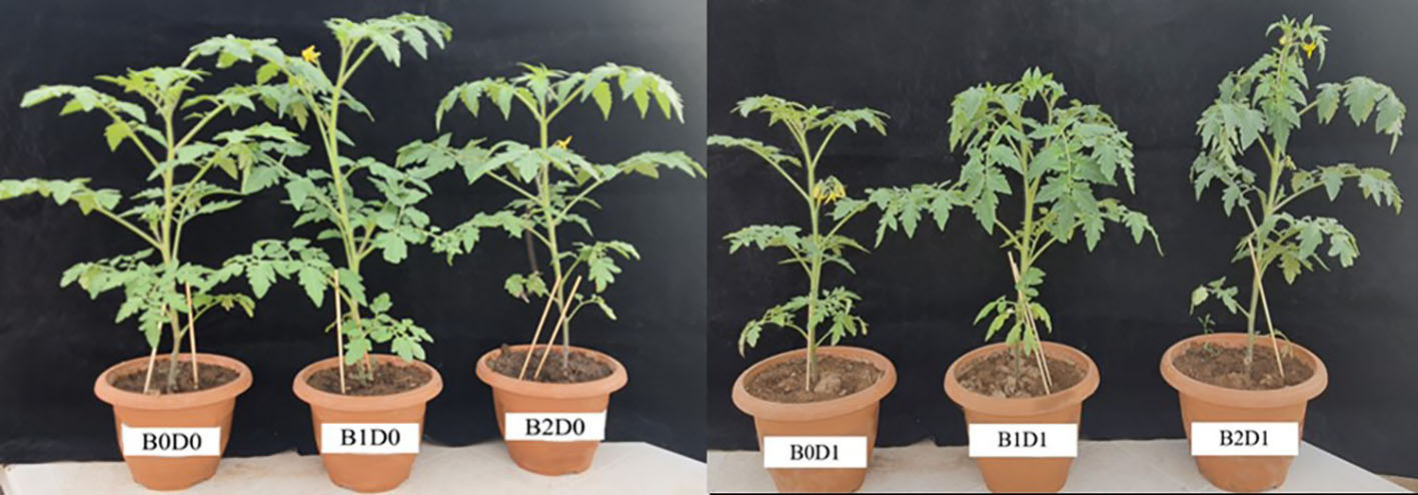
Effect of drought stress and biostimulant applications on plant growth of tomato. B0: Control (no biostimulant), B1: 4 L ha^-1^ biostimulant, B2: 6 L ha^-1^ biostimulant, D0: 100% irrigation (field capacity), D1: 50% irrigation of field capacity.

To clarify the promoting impacts of biostimulants, *Solanum lycopersicum* plants grown in normal and water deficit conditions were treated with biostimulant containing microbial organisms that produce natural hormones such as auxin and gibberellin (*Azotobacter chroococcum, Azosprillium brasilense* (1x10^9^ cfu/ml) combined with natural organic acid, amino acid, and fulvic acid solutions and applied at 4 L ha^-1^ and 6 L ha^-1^ doses. The effects of biostimulant and drought stress on the growth of the tomato seedlings are shown in **Figure 1**.

**Figure 2:**
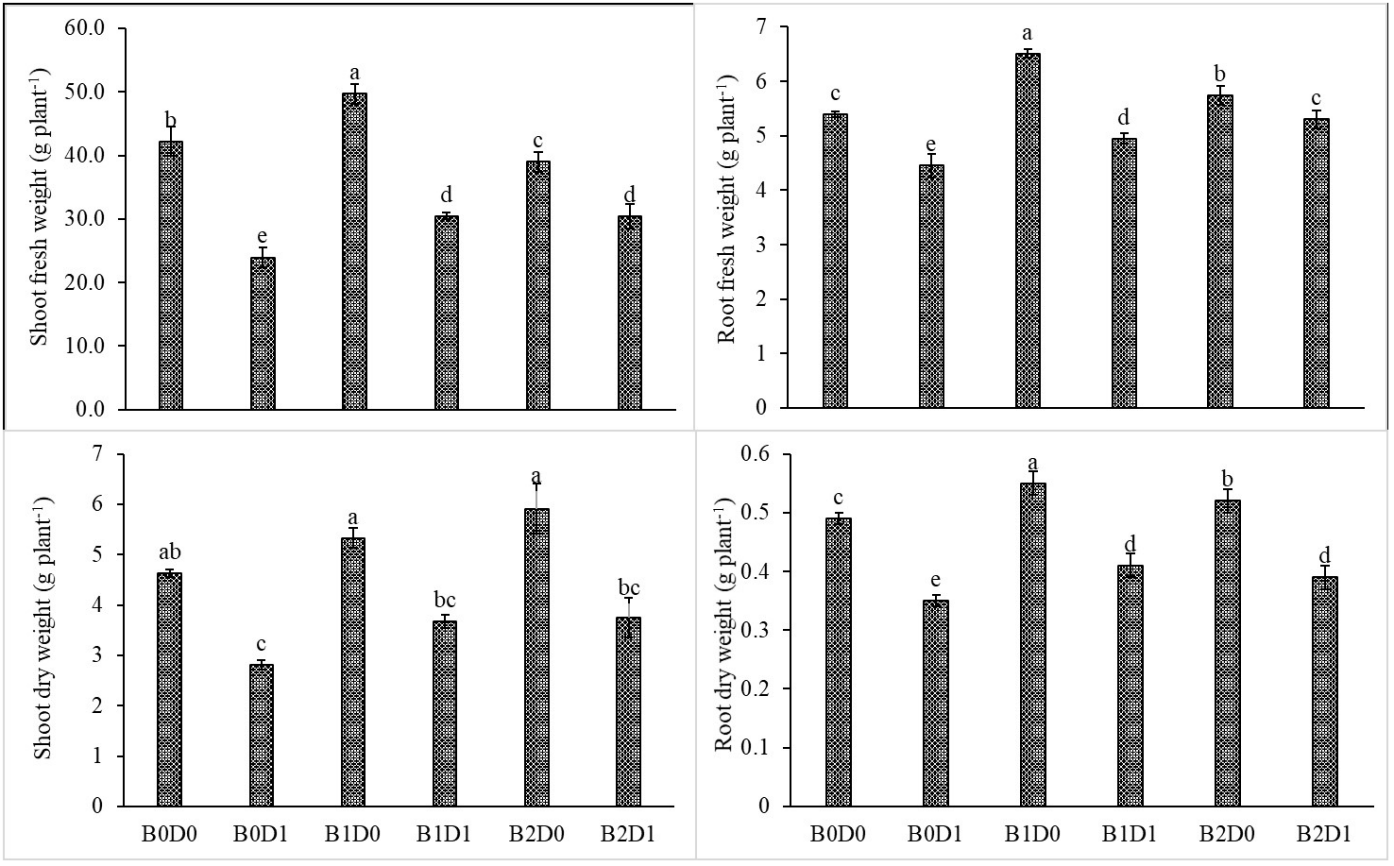
Effect of drought stress and biostimulant applications on shoot fresh weight, root fresh weight, shoot dry weight, and root dry weight of tomato seedlings. The difference between the means indicated by different letters on the bars is statistically significant (Duncan multiple comparison test, P<0,05). B0: Control (no biostimulant), B1: 4 L ha^-1^ biostimulant, B2: 6 L ha^-1^ biostimulant, D0: 100% irrigation (field capacity), D1: 50% irrigation of field capacity.

**Figure 3:**
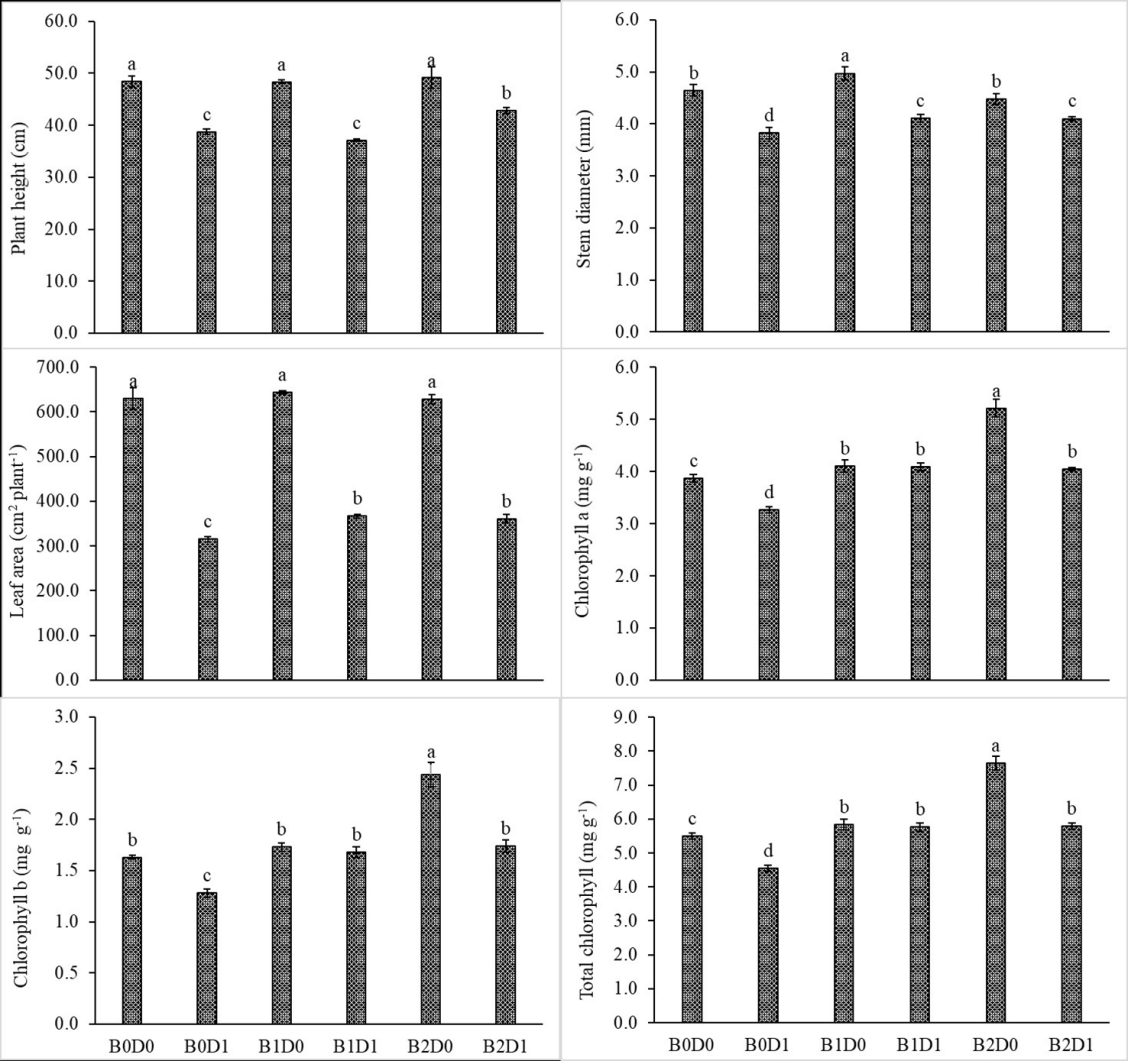
Effect of drought stress and biostimulant applications on plant height, stem diameter, leaf area, chlorophyll a, chlorophyll b, and total chlorophyll of tomato seedlings. The difference between the means indicated by different letters on the bars is statistically significant (Duncan multiple comparison test, P<0,05). B0: Control (no biostimulant), B1: 4 L ha^-1^ biostimulant, B2: 6 L ha^-1^ biostimulant, D0: 100% irrigation (field capacity), D1: 50% irrigation of field capacity.

**Figure 4:**
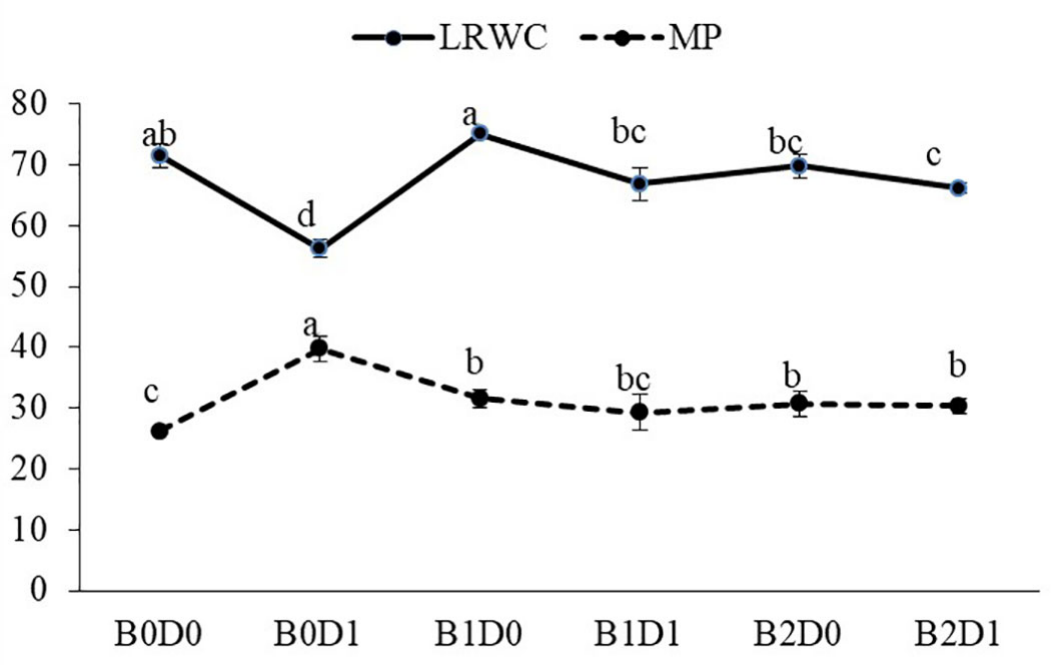
Effect of drought stress and biostimulant applications on leaf relative water content (LRWC) and membrane permeability (MP) of tomato seedlings. The difference between the means indicated by different letters in the same line is statistically significant (Duncan multiple comparison test, P<0,05). B0: Control (no biostimulant), B1: 4 L ha^-1^ biostimulant, B2: 6 L ha^-1^ biostimulant, D0: 100% irrigation (field capacity), D1: 50% irrigation of field capacity.

The results from the analyzed data showed a significant impact from drought stress when using biostimulant on the studied plants. Under drought (B0D1), the shoot fresh weight, root fresh weight, shoot dry weight, root dry weight, plant height, stem diameter, and leaf area decreased by 43%, 19%, 39%, 29%, 20%, 18%, and 50%, respectively, compared to the control (B0D0) (**Figures 2**, **3**). In addition, 21%, 16%, 21%, and 17% reductions occurred in LRWC, Chl a, Chl b, and total chlorophyll contents under drought, respectively (**Figures 3**, **4**). It was detected that although drought stress caused a considerable effect on plant growth, LRWC, Chl a, Chl b, and total chlorophyll, application of biostimulant mitigated its destructive influences significantly. In both B1D1 and B2D1 applications, the decrease of drought stress on these parameters was less compared to B0D1 application. In D1 conditions, the plant morphological growth parameters increased with the B1 and B2 applications compared to B0D1 (**Figures 2**–**4**).

**Figure 5:**
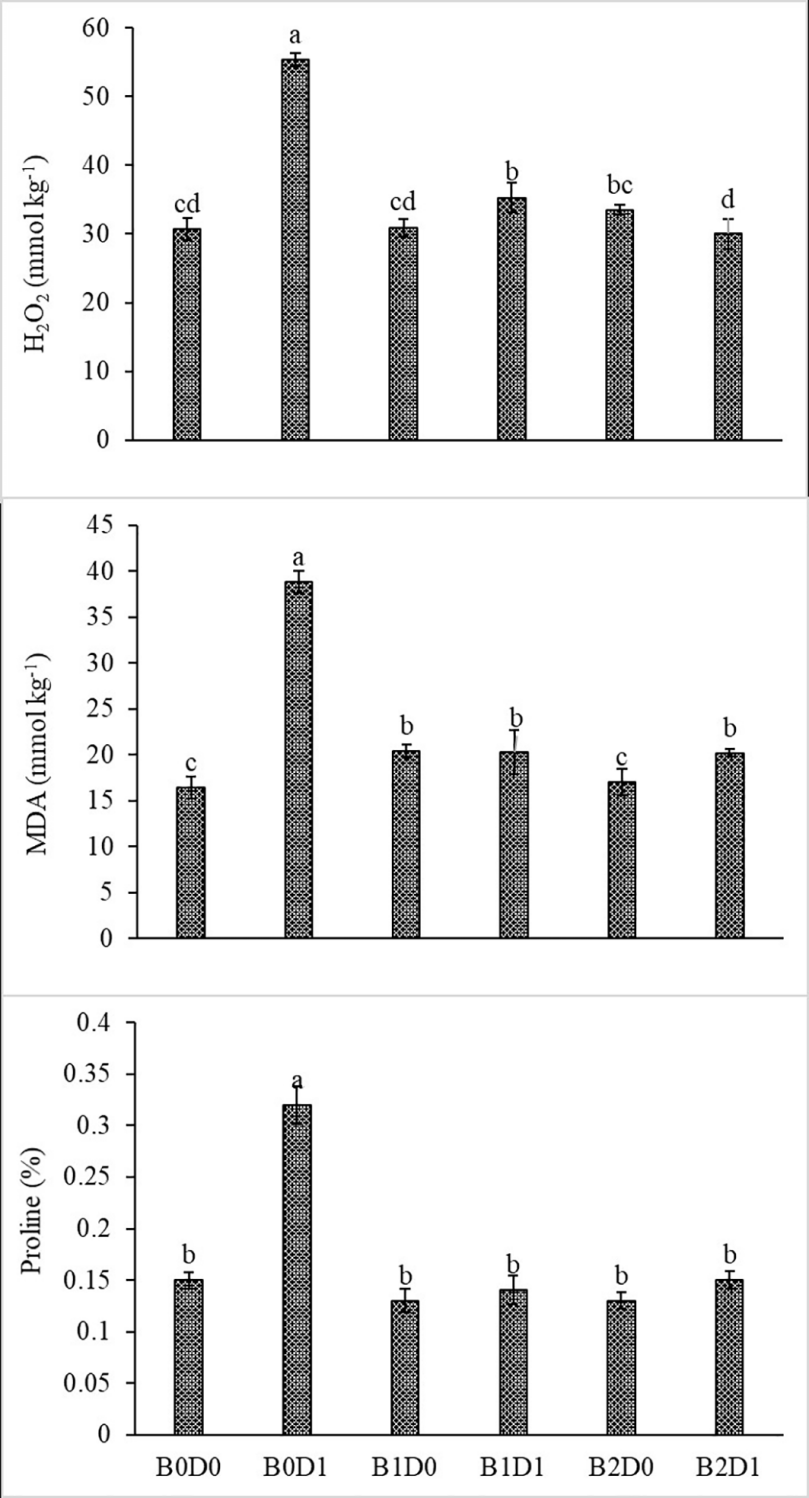
Effect of drought stress and biostimulant applications on H_2_O_2_, MDA, and proline content of tomato seedlings. The difference between the means indicated by different letters on the bars is statistically significant (Duncan multiple comparison test, P<0,05). B0: Control (no biostimulant), B1: 4 L ha^-1^ biostimulant, B2: 6 L ha^-1^ biostimulant, D0: 100% irrigation (field capacity), D1: 50% irrigation of field capacity.

**Figure 6:**
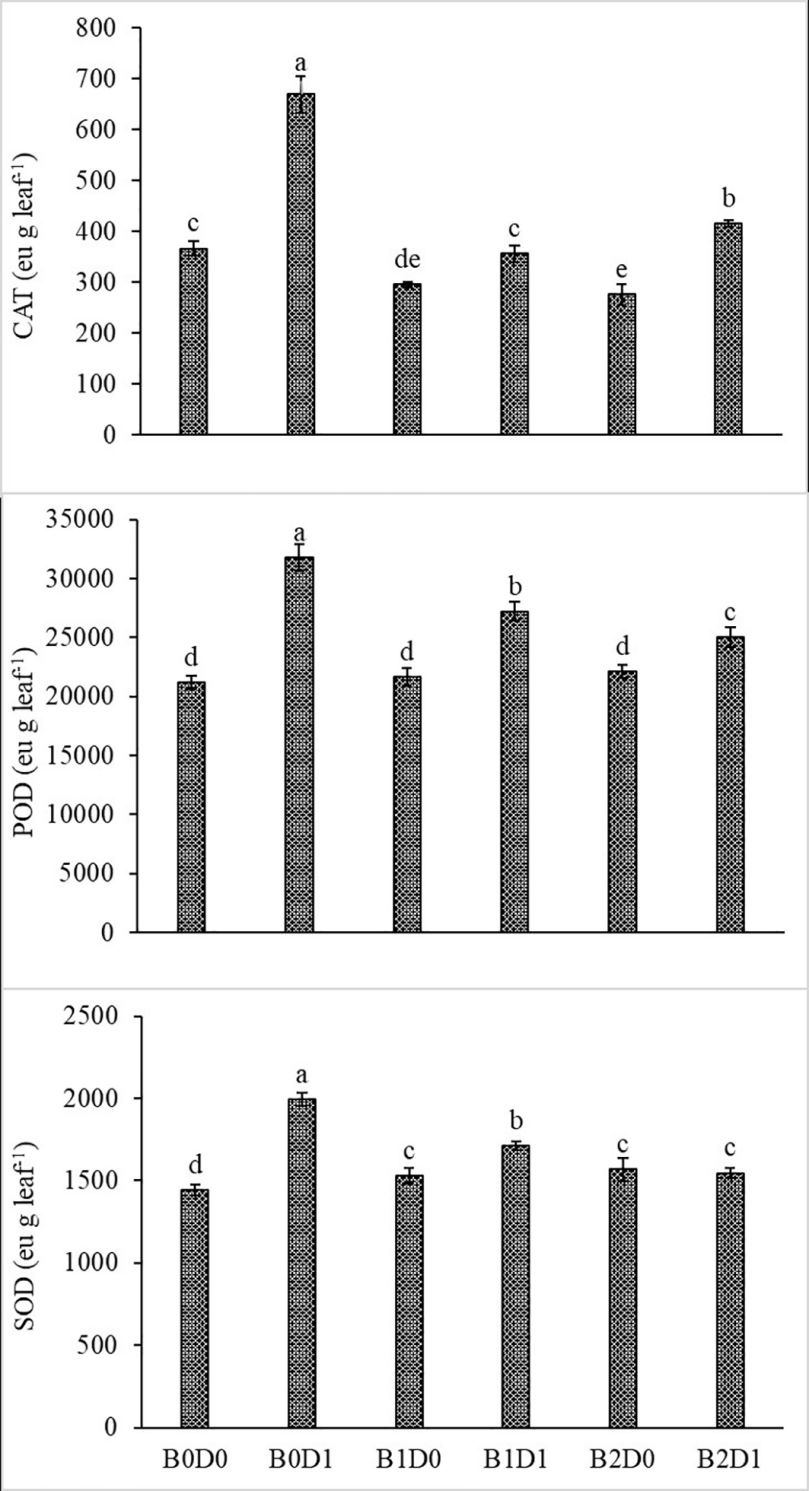
Effect of drought stress and biostimulant applications on CAT, POD, and SOD enzyme activity of tomato seedlings. The difference between the means indicated by different letters on the bars is statistically significant (Duncan multiple comparison test, P<0,05). B0: Control (no biostimulant), B1: 4 L ha^-1^ biostimulant, B2: 6 L ha^-1^ biostimulant, D0: 100% irrigation (field capacity), D1: 50% irrigation of field capacity.

Growing tomato plants in drought-stressed soil stimulated a salient rise in the ROS (H_2_O_2_) levels (**Figure 5**), which catalyzed leakage of ions (MP) (**Figure 4**) and levels of peroxidation of lipids (evaluated as malondialdehyde (MDA) content). Biostimulant treatments were almost equally effective on these parameters (**Figure 5**). The contents of H_2_O_2_ and MDA were also lessened saliently with B1 and B2. In B1D1 and B2D2 applications, H_2_O_2_ content was 36% and 46% lower, and MDA content was 47 and 48% lower, respectively, compared to B0D1 application. Drought stress almost doubled the amount of proline of the tomato seedlings. However, biostimulant treatments lowered the proline content of the tomato seedlings under drought stress conditions (**Figure 5**). Water-restricted conditions resulted in increased antioxidant activity (82%, 50%, and 38% rising in CAT, POD, and SOD, respectively) in the tomato seedlings. Biostimulant-treated tomato seedlings were found to have less CAT, POD, and SOD activity under drought stress but still slightly greater than D0 treatment (**Figure 6**).

The biostimulant application affected the hormone content of the tomato seedlings under both non-drought and drought conditions. Water deficit conditions caused a decrease in the IAA, GA, SA, cytokine, zeatin, and jasmonic acid content of the tomato seedlings by a ratio of 83%, 93%, 82%, 89%, 50%, and 57% compared to the control, respectively. Biostimulant applications exerted statistically significant effects on these hormone contents. In D1 conditions, B2 in terms of IAA, SA, cytokine, and jasmonic acid and B1 application in terms of GA and zeatin gave better results. Drought-stressed tomato seedlings had more ABA content than the non-stressed ones, while B1 and B2 applications decreased ABA content in both drought and normal conditions (**Table 1**).

**Table 1:** Effect of drought stress and biostimulant applications on plant hormone content of tomato seedlings.

Biostimulant	Irrigation	IAA ng mg tissue^-1^	ABA ng g DW^-1^	GA ng g DW^-1^	SA ng g DW^-1^	Cytokinin ng g DW^-1^	Zeatin ng g DW^-1^	Jasmonic acid ng g DW^-1^
B0	D0	2.47 d	975.13 d	6.30 d	8.31 d	5.76 e	2.61 c	20.11 c
	D1	0.42 e	9074.50 a	0.41 e	1.45 f	0.62 f	1.31 d	8.62 d
B1	D0	6.61 b	569.16 e	9.64 c	12.78 b	10.96 b	4.58 a	25.33 b
	D1	3.42 c	3684.58 b	10.88 b	6.20 e	6.72 d	4.42 a	18.35 c
B2	D0	8.92 a	514.50 e	12.60 a	14.01 a	12.88 a	3.38 b	31.26 a
	D1	6.70 b	1151.73 c	10.63 b	10.46 c	8.95 c	3.21 b	21.94 bc

The difference between the means indicated by different letters in the same column is statistically significant (Duncan multiple comparison test, P<0,05). B0: Control (no biostimulant), B1: 4 L ha^-1^ biostimulant, B2: 6 L ha^-1^ biostimulant, D0: 100% irrigation (field capacity), D1: 50% irrigation of field capacity.

Lower irrigation level decreased the soil organic matter and S, Mg, Na, and Zn content (by a ratio of 8%, 14%, 19%, and 38%, respectively), but it did not affect the pH, EC, P, K, Ca, B, Cu, and Mn content. Biostimulant applications at different doses exerted significant effects on some properties of the soil used in the study except for pH and Mn content (**Table 2**). B1 and B2 treatments enhanced OM content of the soil in both water stress (increase of 19% and 16%, respectively) and normal conditions (increase of 50% and 32%, respectively), of which the most effective was B1. Similarly, the total N and P content of the soil was higher in biostimulant applications than the control. In normal or drought soil, supplying tomato plants with biostimulant catalyzed a salient increase in P and K. K content was the highest in B1D0 (58% increase compared to B0D0) followed by B2D0. B1D0, B2D0, and B2D1 elevated the S content of the soil in both drought (39% increase compared to B0D0) and normal (41% increase compared to B0D0) conditions. Compared with the control, the tomato seedlings treated with the biostimulant had increased contents of Ca and Mg by a ratio of 71% and 51%, respectively.

**Table 2:** Effect of drought stress and biostimulant applications on some chemical properties of rhizosphere soil of tomato seedlings.

Bio.	Irrig.	pH	EC (micromhos cm^-1^)	OM (%)	Total N (%)	P (ppm)	K (cmol kg^-1^)	S (mg kg^-1^)	Ca (mg kg^-1^)	Mg (cmol kg^-1^)	Na (cmol kg^-1^)	B (ppm)	Cu (ppm)	Fe (ppm)	Zn (ppm)	Mn (ppm)
B0	D0	7.08 ns	58.16 b	1.17 d	0.03 c	23.19 d	1.53 c	7.81 c	7.18 c	5.33 d	1.67 b	0.01 b	0.07cd	0.36 ab	0.08 ab	0.05ns
	D1	7.05	55.02 b	1.08 e	0.03 c	22.24 d	1.49 c	6.72 d	7.24 c	4.50 e	1.35 c	0.01 b	0.06 d	0.32 bc	0.05 d	0.04
B1	D0	7.12	71.99 a	1.75 a	0.06 a	48.02 a	2.42 a	10.18 b	12.26 a	8.07 a	1.95 a	0.01 b	0.10 a	0.33 ab	0.07bc	0.05
	D1	7.01	49.63 b	1.39 c	0.05 a	38.91 c	1.51 c	8.23 c	8.66 b	4.94 d	1.49 bc	0.02 b	0.06 d	0.27 c	0.06 c	0.05
B2	D0	7.13	55.33 b	1.55 b	0.06 a	44.85 b	1.81 b	10.98 a	8.35 b	7.20 b	1.68 b	0.03 a	0.09 ab	0.38 a	0.09 a	0.06
	D1	7.22	62.20 ab	1.36 c	0.04 b	44.85 b	1.58 c	10.05 b	8.22 b	6.54 c	1.51 bc	0.02 b	0.08 bc	0.28 c	0.07bc	0.04

The difference between the means indicated by different letters in the same column is statistically significant (Duncan multiple comparison test, P<0,05). B0: Control (no biostimulant), B1: 4 L ha^-1^ biostimulant, B2: 6 L ha^-1^ biostimulant, D0: 100% irrigation (field capacity), D1: 50% irrigation of field capacity.

## 4 Discussion

Drought is one of the leading factors that cause serious yield losses in agricultural production (Wahab et al., 2022). The duration of a water shortage is critical to plant growth and thus the crop yield. In the first periods of drought conditions, root development is triggered in order to reach more water while stem elongation is slowed down. If water-deficient conditions prolong, both stem and root development stop, the leaf area and number of leaves decrease, and leaves may turn yellow and fall off. Our study showed that biostimulant application decreased the shoot fresh weight, root fresh weight, shoot dry weight, root dry weight, plant height, stem diameter and leaf area by 43%, 19%, 39%, 29%, 20%, 18% and 50%, respectively, compared to the control (B0D0) (**Figures 2**, **3**). In addition, 21%, 16%, 21%, and 17% reductions occurred in the LRWC, Chl a, Chl b, and total chlorophyll contents under drought, respectively (**Figures 3**, **4**).

When the B1D0 application was compared with the B0D0 application, an increase of 18%, 21%, 15%, 12%, 7%, and 2% occurred in the shoot fresh weight, root fresh weight, shoot dry weight, root dry weight, stem diameter, and leaf area, respectively. Similar to the results of previous studies (Hao et al., 2019; Liang et al., 2020; Nephali et al., 2020; Lamin-Samu et al., 2021; Ors et al., 2021; Gedeon et al., 2022), our findings showed that water deficit conditions negatively affected the growth of tomato seedlings (**Figures 1**, **2**). The decrease in the plant growth resulted from the cessation of cell division and expansion of cells in shoot and root meristems, reduction in chlorophyll content, degradation of chloroplast structure, reduction in electron transport rate (ETR), downregulation of photosynthesis rate, degradation of D1 protein, and decrease in quantum yield of photosystem II and efficiency due to the decrease in the rate of photosynthesis as a result of water deficiency (Anjum et al., 2011a; Wang et al., 2019; Yang et al., 2019; Nagamalla et al., 2021; Zhang et al., 2021).

Biostimulants play an important role in “Induced Systemic Tolerance” (IST), which involves the physical and chemical changes that provide tolerance to abiotic stresses in plants (Bulgari et al., 2019; Turan et al., 2021). Under drought stress, the plant with damaged physiology alters its water relation to maintain cellular functions and accumulates a range of osmotically active molecules/ions including soluble sugars, sugar alcohols, and amino acids (Chen et al., 2020; Ozturk et al., 2021). The tolerance against drought stress and water use efficiency could be improved by using biostimulants such as PGPR, fulvic acid, and amino acids. Earlier studies reported that biostimulants containing PGPR improve plant growth in arid or semi-arid conditions (Heidari and Golpayegani, 2012; Gururani et al., 2013), which might be explained by root development in plants due to IAA and GA production by bacteria (Sahin et al., 2015; Ekinci et al., 2021). Fulvic acids were also shown to increase plant growth by improving photosynthesis, respiration rate, intercellular CO_2_ concentration, and proline content with and without drought conditions (Anjum et al., 2011b; Wang et al., 2023). Amino acids play a supportive role in the resistance and defense mechanisms of plants against abiotic stress factors and oxidative conditions. Glycine, betaine, and proline are known to remove ROS and stimulate stress-related genes (Abdelaal et al., 2021; Kayak et al., 2023). Proline and some other osmoprotectants contribute to the regulation of many vital processes, such as protein and enzyme stabilization, storage of metabolic energy, osmoregulation, osmoprotection, and signal transduction, in plants under some abiotic stress conditions such as drought (Zulfiqar et al., 2020; Zulfiqar and Ashraf, 2022). Lee et al. (2006) stated that arginine is effective in N storage and transport under biotic and abiotic stress conditions. With glutamate applications, primary root development slows down, and root branching increases, since glutamate plays a signaling role in plants for the roots to develop into parts of the soil where nutrients are more available (Walch-Liu et al., 2006a; Walch-Liu et al., 2006b; Forde and Lea, 2007).

The decrease in the chlorophyll content of tomato plants that are subjected to drought stress (**Figure 3**) might be explained by the activity of chlorophyllase (chl-degrading enzyme) and the inhibition of chlorophyll biosynthesis after increased production of ethylene due to formation of ROS such as singlet oxygen, superoxide anion, and H_2_O_2_ (Sachdev et al., 2021). As can be seen in **Figure 5**, drought stress increased H_2_O_2_ and MDA content due to the decrease in the photosynthesis rate.

As predicted, drought caused a reduction in the LRWC of the tomato seedlings (**Figure 4**) since the first response to water stress is to decrease the tissue water content. In particular, drought stress has been reported to reduce the leaf water potential (Tuver et al., 2022). Both ionic imbalance and osmotic stress under drought stress could be linked to the reduction in water content (RWC) (Rady et al., 2021). As expected, the MP of the plant was found to increase with drought stress. The MP values were the lowest under well-watered conditions and significantly increased under water deficiency (**Figure 4**). The major metabolic damage caused by drought stress was reported as the membrane damage resulting in increased lipid peroxidation (Liang et al., 2020). The production of ROS is an obvious consequence of abiotic stresses and is gaining importance, not only because of their ubiquity in plants and their subsequent deleterious effects but also for their diversified roles in the signal chain influencing other biomolecules, hormones involved in growth, development, or stress regulation (Hasanuzzaman et al., 2020). Biostimulant amendment to soil enhanced chlorophyll content while it reduced H_2_O_2_ and MDA content and MP (**Figures 4**, **5**). The biostimulant used in this study directly and positively influenced the plant response to drought, with a persisting effect until the end of the water-deficient conditions. The biostimulant-treated plants were able to use the scarce water resource more efficiently.

**Figure 5** shows that proline content increased in plants subjected to drought stress. Proline acts as an osmoprotectant and directly stabilizes proteins, membranes, and other subcellular structures, scavenges free radicals, and balances the cell redox status under stress conditions (Abdelaal et al., 2022). Drought changes the plant cell turgor pressure (i.e., the amount of water potential), and thus osmotic balancing is crucial to ensure that plant cells are least affected by water stress.

The activity of CAT, POD, and SOD increased with water deficiency (**Figure 6**). Previous studies also reported increased antioxidant enzyme (SOD, CAT, and POD) activities in tomato (*Solanum lycopersicum* L.) under drought (Yuan et al., 2016; Hao et al., 2019; Liang et al., 2020). Antioxidant enzyme activity plays an important role in increasing tolerance to drought stress in plants. This can be achieved by reducing the negative effects of free radicals, which increase especially under stress conditions. Reduction of reactive oxygen compounds formed during drought stress and prevention of their accumulation are important factors in the struggle of plants with stress conditions. In the fight against oxidative stress caused by the accumulation of reactive oxygen compounds, plants use enzymatic (SOD, POD, CAT, and APX) or non-enzymatic (glutathione, ascorbate, tocopherols, and carotenoids) antioxidant molecules. While the main task of non-enzymatic antioxidant molecules is to protect photosynthetic membranes, enzymatic antioxidant molecules prevent their accumulation by reducing reactive oxygen compounds (Impa et al., 2012). Biostimulant treatments in water deficit conditions modulate the antioxidant enzyme activities compared with stressed untreated plants. Protein–protein interactions of antioxidant enzymes represent an important part of the machinery of ROS regulation. Through protein–protein interactions, these enzymes are regulated by folding, stabilization, degradation, and activation, which have crucial consequences in ROS accumulation and plant stress tolerance. On the other hand, protein–protein interactions may link ROS scavenging with diverse metabolic and physiological processes (Mittler et al., 2022).

These impacts on stressed plants were confirmed by Turan et al. (2021). Biostimulants might have reduced the negative effects of drought stress on the tomato seedlings by improving soil structure and enhancing organic matter with humic and fulvic substances. Drought stress led to damage to the plant physiology with oxidative stress induction by generating ROS such as hydroxyl radicals, singlet oxygen and H_2_O_2_, membrane damage, and altered antioxidant enzymatic activity, leading to the loss of membrane integrity and damage to the ATP synthase. Moreover, biostimulant applications lowered CAT, POD, and SOD activity of the tomato seedlings under water deficit conditions (**Figure 6**), which is also in agreement with previous studies (Vasconcelos et al., 2009; Kałużewicz et al., 2017; Rady et al., 2020; Agliassa et al., 2021; Rai-Kalal et al., 2021).

Lower irrigation levels caused a reduction in IAA, GA, and SA content but increased ABA content (**Table 1**). Under drought stress, there is a decrease in the stomatal openings in leaves (Decoteau, 2000), which is associated with a decrease in the level of intrinsic cytokinins and an increase in the level of ABA. In an earlier study, ABA levels were found to increase, while auxin, gibberellin, and cytokinin levels decreased in corn plant (*Zea maize*) grown in arid conditions, and this could be reversed with biostimulant applications. This might be explained by the antagonistic relationship due to the common biosynthetic origin of cytokinins with ABA. In plants exposed to drought stress, the amount of ABA increases in stomatal cells; as a result, water-insoluble starch is formed, and K ion decreases (Nelissen et al., 2018). Drought stress also affects the production of the endogenous level of hormones, such as ABA, jasmonic acid (JA), ethylene, GA, auxins, SA, and cytokinins (Ullah et al., 2018). ABA synthesis is one of the fastest responses of plants to drought stress causing stomatal closure. In addition, SA is involved in the regulation of drought responses, enhancing antioxidant enzymatic activities together with other physio-biochemical traits (Liu et al., 2022). In our experiment, while endogenous ABA levels in stressed plants increased, jasmonic acid decreased compared to the control plants (**Table 1**). The active derivative of jasmonic acid, also known as jasmonates, has an important role in controlling the response to various biotic and abiotic stresses (Ullah et al., 2018). Mia et al. (2012) suggested that growth-promoting effects of PGPR on plants could be attributed to the production of hormones. The present research shows that biostimulant treatment elevated the IAA, GA, SA, cytokinine, zeatin, and jasmonic acid content under lower irrigation levels, which shows its particular ability to stimulate plant growth under abiotic stress conditions. The PGPR strains used in this study might have affected the root hormone concentrations by producing plant hormones in the rhizosphere, which were then absorbed by the root (Turan et al., 2014; Arkhipova et al., 2020). In mung beans (*Vigna radiata*) exposed to water stress, it has been reported that the IAA level increased with the application of drought-tolerant *Pseudomonas aeruginosa* GGRJ21 (Sarma and Saikia, 2014). PGPR administration can be effective in the formation of a tolerance mechanism developed with stress by increasing cytokinin and IAA levels (Kudoyarova et al., 2019).

The findings of the study showed that lower irrigation level caused a decrease in S, Mg, Na, and Zn but did not statistically affect the pH, EC, total N, K, Ca, B, Cu, Fe, and Mn content of the experiment soil (**Table 2**). Decreases in nutrient uptake during drought occur due to reduced mineralization, nutrient diffusion, and mass flow in the soil. Drought also decreases nutrient uptake by affecting the kinetics of nutrient uptake by the roots (Martinez et al., 2020).

Furthermore, biostimulant applications increased OM and total N, P, K, S, Ca, Mg, and Fe content under both normal and drought conditions (**Table 2**). They also stimulated the working of microorganisms as activators, non-symbiotic N fixation, enzyme increase in plant nutrient solubility, siderophore production, solubility of mineral nutrients of the soil, and increase of the nutrient uptake and production of volatile organic compounds. In addition to their role in N fixation of the aforementioned bacteria, they also have been shown to synthesize vitamins such as thiamine and riboflavin and hormones such as auxin, gibberellin, and cytokinin (Abd El-Fattah et al., 2013). It has been reported by different researchers that bacteria convert P in the soil into receivable forms. The mechanisms most used by microorganisms to dissolve P are the production of organic acids (Goldstein, 1995; Basu et al., 2021) and production of phosphatases (Rodríguez et al., 2006; Turan et al., 2021; Da Silva et al., 2023).

These effects of biostimulant applications on the soil are also due to the humic and fulvic acids it contains. Fulvic acids can adsorb more cations by having a higher cation exchange capacity than humic acids due to their higher content of carboxyl groups (Bocanegra et al., 2006). Due to their small molecular size, fulvic acids can pass through biological membranes. Fulvic acids have positive effects on the availability and transport of Fe and other microelements due to both their chelating properties and their ability to pass through cell membranes (Bocanegra et al., 2006; Turan et al., 2022). Fulvic acids also have positive effects on root growth (Lulakis and Petsas, 1995; Dobbss et al., 2007). In addition, positive effects of fulvic acid applications on root organs have been observed.

Humic substances increase the cation exchange capacity (CEC) of soils and increase soil fertility. Humic acids contribute to plant growth and development by improving the structure of the soil. Humic acids slow down the evaporation of water in the soil. This is especially important for increasing the water-holding capacity of soils with little or no clay. Since humic and fulvic acids have colloidal properties, they increase aggregate formation by binding sand, silt, and clay fractions and improve soil structure. Humic acids stay in the soil for a long time and are gradually broken down over time. In addition to the positive effect of humic substances on soil properties, it also directly contributes to the plant with the plant nutrients released at the end of mineralization (Ampong et al., 2022).

## 5 Conclusions

To improve abiotic stress tolerance, the roles of biostimulants have been investigated in various plant species by different studies. Our findings showed that the application of biostimulants is a promising strategy to alleviate the negative impacts of drought stress on the growth of tomato seedlings under drought condition. It is thought that with the biostimulant formulations used in the study, the physiological problems caused by drought stress in the plant and the consequent yield losses can be reduced. In order to determine this more clearly, it would be beneficial to take this study one step further and move on to fruit yield.

## Data availability statement

The raw data supporting the conclusions of this article will be made available by the authors, without undue reservation.

## Author contributions

MT, ME, and EY designed and conducted the study, collected the data, and performed the analysis and statistical tests of the experiments. MT, ME, SA, MB, and EY wrote and contributed to the manuscript and approved the submitted version.
